# Long Non-Coding Ribonucleic Acid Taurine Up-Regulated Gene 1 Accelerates Ventricular Septal Defect Formation in Children

**DOI:** 10.21470/1678-9741-2025-0181

**Published:** 2026-09-10

**Authors:** XiaoMei Zhong, Wei Jin, LiHong He, JieJing Liang

**Affiliations:** 1 Department of Pediatrics, Ganzhou People's Hospital, Ganzhou City, Jiangxi Province, People’s Republic of China; 2 Department of Pediatrics, Suizhou Central Hospital (Affiliated Suizhou Hospital of Hubei University of Medicine), Suizhou City, Hubei Province, People’s Republic of China; 3 Department of Outpatient, The General Hospital of the PLA Western Theater Command, Chengdu City, Sichuan Province, People’s Republic of China; 4 Department of Pediatrics, Heilongjiang Provincial Hospital, Harbin City, Heilongjiang Province, People’s Republic of China

**Keywords:** Ventricular Septal Defect, TUG1, HIF1AN, miR-222-3p, Cardiomyocytes.

## Abstract

**Introduction:**

This study explored the function and molecular mechanism of long non-coding ribonucleic acid taurine up-regulated gene 1 (TUG1) in children with ventricular septal defect (VSD).

**Methods:**

To establish an in vivo VSD model, 10 pregnant mice were administered valproic acid via intraperitoneal injection (VSD group, n = 10). Another 10 pregnant mice were used as the control group and administered 0.9% sodium chloride solution via intraperitoneal injection. Subsequently, the cardiac function and cardiac tissue histopathological characteristics in both groups were evaluated. TUG1, miR-222-3p, and hypoxia-inducible factor 1-alpha subunit inhibitor (HIF1AN) expression levels in cardiac tissues were tested. P19 cells were chosen to simulate VSD in vitro model, and the cell progression was tested. P19 cells were induced to differentiate into cardiomyocytes with dimethyl sulfoxide, and test of the shape and beating frequency of cardiomyocytes was conducted. Left ventricular fractional shortening, ejection fraction, and systolic and diastolic thickness of the cardiac anterior wall were significantly reduced in VSD fetal mice. VSD formation, aortic overlay, and dysplasia were observed.

**Results:**

TUG1 and HIF1AN were upregulated, and miR-222-3p was downregulated in VSD mice. Silencing TUG1 or HIF1AN or elevating miR-222-3p facilitated P19 cell progression and differentiation, whereas lowering miR-222-3p did the opposite. TUG1 was a molecular sponge of miR-222-3p, which targeted HIF1AN. Elevating HIF1AN reversed the impacts of silencing TUG1 or elevating miR-222-3p on P19 cells.

**Conclusions:**

The findings of this study could offer a new insight into the molecular basis of VSD and lay the groundwork for new diagnostic techniques.

## INTRODUCTION

**Table:** 

Abbreviations, Acronyms & Symbols				
Ago2 ANOVA	= Argonaute-2 = Analysis of variance		MTT	= 3-(4,5-dimethylthiazol-2-yl)-2,5-diphenyltetrazolium bromide
APC-H	= Allophycocyanin-height		NCs	= Negative controls
cDNA	= Complementary deoxyribonucleic acid		OD	= Optical density
CHD	= Congenital heart defect		pcDNA	= Plasmid cloning deoxyribonucleic acid
cTnT	= Cardiac troponin T		PE-H	= Phycoerythrin-height
D	= Day of pregnancy		PM	= Particulate matter
DMSO	= Dimethyl sulfoxide		RIP	= Ribonucleic acid immunoprecipitation
EBs	= Embryoid bodies		RNA	= Ribonucleic acid
FBS GAPDH	= Fetal bovine serum = Glyceraldehyde-3-phosphate dehydrogenase		RT-qPCR	= Reverse transcription-quantitative polymerase chain reaction
HIF-1α	= Hypoxia-inducible factor 1-alpha		sh-NC	= Short hairpin ribonucleic acid negative control
HIF1AN	= Hypoxia-inducible factor 1-alpha subunit inhibitor		shRNA	= Short hairpin ribonucleic acid
IgG LncRNA	= Immunoglobulin G = Long non-coding ribonucleic acid		sh-TUG1	= Short hairpin ribonucleic acid targeting taurine up-regulated gene 1
LV	= Left ventricular		TUG1	= Taurine up-regulated gene 1
LVAWTd	= Left ventricular anterior wall thickness at diastole		VPA	= Valproic acid
LVAWTs	= Left ventricular anterior wall thickness at systole		VSD	= Ventricular septal defect
α-MEM	= Minimum essential medium, alpha modification			
mRNA	= Messenger ribonucleic acid			

A congenital heart defect (CHD) known as ventricular septal defect (VSD) involves a hole in the partition between the right and left ventricles^[^1^]^. VSD cases are usually asymptomatic. Techniques such as echocardiography are being used more frequently to screen for VSD, which has become much more common in neonates^[^2^]^. However, the pathogenesis and molecular mechanisms of VSD are not completely understood, and further research is urgently needed.

Long non-coding ribonucleic acids (LncRNAs) have attracted much attention for their role in modulating the expression or function of protein-coding genes. They are involved in a range of cellular biological functions, such as cell growth, differentiation, and apoptosis^[^3^]^. LncRNA SNHG6 participates in the formation of VSD by negatively modulating miR-101 and activating the Wnt/β-catenin pathway^[^4^]^. LncRNA uc.167 impacts the proliferation, apoptosis, and differentiation of P19 cells by modulating Mef2c in VSD^[^5^]^. Taurine up-regulated gene 1 (TUG1) is a lncRNA that has been studied in cancer^[^6^]^, cerebral ischemia/reperfusion injury^[^7^]^, and cardiac vascular disease^[^8^]^. However, the expression and function of TUG1 in VSD remain uncertain.

miRNAs are small, single-stranded ribonucleic acid (RNA) sequences that do not encode proteins and affect gene expression by binding to the 3'-UTRs of messenger RNAs post-transcriptionally^[^9^]^. miRNAs contribute significantly to the homeostasis of the cardiovascular system and are viewed as fine-tuners of cardiac development^[^10^]^. According to Li et al.^[^11^]^, 36 miRNAs, including hsa-miR-222-3p, are dysregulated in patients with VSD. MiR-222-3p shows reduced expression in individuals with VSD^[^11^]^, yet its function in this condition is not well understood.

This study aimed to figure out the function and molecular mechanism of TUG1 in VSD. Through *in vitro* and *in vivo* experiments, the underlying mechanism of TUG1 in VSD and its interaction with miR-222-3p were unveiled. These findings could assist in VSD diagnosis and treatment.

## METHODS

### Ethics Statement

All animal care and experimental studies were conducted following the Code and Guidelines for the Care and Use of Laboratory Animals by the Research Ethics Committee of Ganzhou People's Hospital. All procedures strictly adhered to the ARRIVE guidelines. This study was approved by the Ethics Committee of Ganzhou People's Hospital (TY-ZKY2024-166-01). All procedures comply with the guidelines for the use of laboratory animals of the National Institutes of Health.

### Animal Grouping and Model Establishment

Exposure to valproic acid (VPA) during pregnancy results in congenital heart disease^[^12^]^. Forty mice, healthy and clean, were supplied by Shanghai SLAC Laboratory Animal Co., Ltd. (0042561; body weight: 25 ± 30 g; age: 7 ± 9 weeks). They were placed in cages with a 3:1 ratio of females to males. The first day of pregnancy (D1) was recorded as the date the vaginal plug was found. On D7, 20 female mice were selected, 10 of which were injected intraperitoneally with VPA (H20010595, Guangdong Kangkaiduo Chain Pharmaceutical Co., Ltd.) at 700 mg/kg as the VSD group. The other 10 mice were injected intraperitoneally with 0.9% NaCl as the control group^[^13^]^.

### Cardiac Function Measurement

Pregnant mice were anesthetized and their embryonic hearts were examined by M-mode echocardiography using a Vevo 2100 ultrasound system equipped with an MS 700 transducer (VisualSonic, Toronto, Ontario, Canada)^[^14^]^. An incision was made in the abdominal wall to expose the uterine sac encasing the fetus. Using short-axis M-mode images, left ventricular (LV) diameters at end-diastole and end-systole were assessed, and LV ejection fraction along with fractional shortening were derived.

### Hematoxylin and Eosin Staining

On D19^[^15^]^, both the VSD and control groups of mice experienced a uterine incision delivery, and fetal hearts were collected from each group (n = 10 per group). Fetal cardiac tissues were fixed in 4% formalin-phosphate buffered saline, followed by dehydration (alcohol concentration increased from 60% to 100%), xylene clearance, and paraffin embedding for histological studies. Paraffin sections (4 mm thickness) were stained with hematoxylin and eosin. Images were taken with an Olympus U-CMAD3 camera after observing sections under an Olympus SZX12 inverted microscope^[^16^]^.

### Immunohistochemistry Analysis

Cardiac specimens were fixed with 4% paraformaldehyde and embedded in paraffin. The specimens were cut into 5 µm thick sections and stained with anti-hypoxia-inducible factor 1-alpha subunit inhibitor (HIF1AN) primary antibody (1:1000, NB100-428, Novus Biologicals) and secondary antibody (1:500). The sections were then stained with diaminobenzidine, and images were taken with a fluorescence microscope (Nikon 80i).

### Induction of Differentiation of P19 Cells

P19 cell line (American Type Culture Collection, Manassas, Virginia, United States of America) was cultured in minimum essential medium, alpha modification (α-MEM) supplemented with 10% fetal bovine serum (FBS), 100 U/ml penicillin, 100 mg/ml streptomycin, and 1% dimethyl sulfoxide (DMSO) (Sigma, United States of America) for four days. The cells were cultured into embryoid bodies (EBs) in 6 cm dishes with a complete medium for eight days. Cells were harvested on days zero, four, eight, 10, and 12 of the differentiation process. Using a phase contrast inverted microscope (Nikon Eclipse TE300) equipped with a Nikon E4500 digital camera, morphological changes were photographed. Cardiac troponin T (cTnT) and Nkx2.5 during differentiation were detected using Western Blot^[^17^]^. The plaque contraction phenotype of differentiated cells in EB outgrowth was recorded through video. The specified areas were recorded continuously in real-time for around 25 minutes using a JVC video cassette recorder and SP-mode VHS videotape^[^18^]^.

### Cell Transfection

miR-222-3p mimic/inhibitor, si-HIF1AN, and corresponding negative controls (NCs) were purchased from RiboBio (Guangzhou, China), and short hairpin RNA (shRNA) sequences targeting TUG1 and sh-NC were obtained from GenePharma (Shanghai, China). An overexpression vector for HIF1AN was developed by cloning its complementary deoxyribonucleic acid (cDNA) into the pcDNA™3.1 vector (Invitrogen). Transient transfection of cells was performed with RNAiMax with Plus reagent (Thermo Fisher Scientific) and Lipofectamine 3000^[^19^]^.

### 3-(4,5-Dimethylthiazol-2-yl)-2,5-Diphenyltetrazolium Bromide Assay

Cardiomyocytes, at 1 × 10^7^ cells/L, were seeded in a 96-well culture plate at 200 µL/well and added with 3-(4,5-dimethylthiazol-2-yl)-2,5-diphenyltetrazolium bromide (or MTT) solution. After centrifugation, the culture medium was removed, and 0.15 mL DMSO (Deyan, Shandong, China) was added. Then absorbance (A) value was measured with a microplate reader (SAFAS Xenius XL, Shanghai Ruixuan) (detection wavelength of 570 nm).

### Cell Cycle Detection

P19 cells were stained using a cycle assay and deoxyribonucleic acid kit (BD Biosciences, Franklin Lakes, New Jersey, United States of America) and quantified using a flow cytometer (BD Corporation). The proportion of cells in G0/1, S and G2/M phases was analyzed using ModFit software (version 5.0)^[^20^]^.

### Cell Migration Assay

Cell migration ability was assayed by the Transwell assay. P19 cells underwent incubation with 0.25% trypsin/ethylenediaminetetraacetic acid, were then reconstituted to a concentration of 5 × 104 cells/ml in serum-free α-MEM, and a 100 µl cell suspension was placed in the upper cell chamber (BD Bioscience, New York, Unites States of America). Meanwhile, 600 µl of culture medium with 10% FBS was covered on the lower chamber. Post-incubation, cells failing to traverse the membrane were cleansed using a cotton swab, while those infiltrating the lower membrane were stabilized with 4% formaldehyde and colored with a crystal violet solution. A minimum of five fields of view, chosen at random from each well, were captured using a light microscope^[^21^]^.

### Apoptosis

P19 cells were placed into 6-well plates with 2 × 10^4^ cells per well, followed by transfection. After removing the medium, 0.5 ml paraformaldehyde was added to each well, and then the medium was removed. Each well was stained with 0.5 ml Hoechst 33258 (Haimen Biotek Biotechnology Institute, China). Fluorescence analysis was performed under a fluorescence microscope at × 100.

P19 cells were detached with trypsin and double-stained with AnnexinV fluorescein isothiocyanate/propidium iodide. Apoptosis was detected using a BD FACSCalibur (Becton Dickinson, New Jersey, United States of America) flow cytometer.

### Quantitative Polymerase Chain Reaction

Total RNA extraction from tissues and cells was performed with TRIzol reagent (Thermo Fisher Scientific, Inc.). cDNA was produced by reverse transcription using the All-in-One TM First Strand cDNA Synthesis Kit (GeneCopoeia, Guangzhou, China). The activity of relevant genes was verified using QuantiFast® SYBR® Green PCR kit (Qiagen). Comparative quantitative evaluation was performed by 2-∆∆Ct technique. The primers were produced by Shanghai Sangon. The primer sequences are shown in Table 1.

**Table 1:** Reverse transcription-quantitative polymerase chain reaction primer sequence.

Genes	Forward (5’-3’)	Reverse (5’-3’)
TUG1	GGCACCCAGTGTAAAGCA	AAGCAGCAGATAACAGAGTTG
HIF1AN	TGCAGCAAACACTCAATGACACCG	AGTGAGCAGGTGTCACATTCCCTT
GAPDH	GTCAACGGATTTGGTCTGTATT	AGTCTTCTGG GTGGCAGT
MiR-222-3p	ACACTCCAGCTGGGAGCTACATCTGG	CTCAACTGGTGTCGTGGA
U6	CGCTTCGGCAGCACATATAC	TTCACGAATTTGCGTGTCATC

GAPDH=glyceraldehyde-3-phosphate dehydrogenase; HIF1AN=hypoxia-inducible factor 1-alpha subunit inhibitor; TUG1=taurine up-regulated gene 1

### Western Blot

Total protein was harvested utilizing a radioimmunoprecipitation buffer (Solarbio, Beijing, China). The analysis of protein samples utilized the bicinchoninic acid protein Assay Kit (Solarbio), with 20 µg samples undergoing separation via sodium dodecyl sulfate-polyacrylamide gel electrophoresis. Proteins were electro-blotted onto polyvinylidene fluoride membranes (Millipore, Massachusetts, United States of America), blocked with 5% skimmed milk, and incubated with primary antibodies HIF1AN (1: 1000, NB100-428, Novus Biologicals), cTnT (1: 3000; ab8295; Abcam), and Nkx2.5 (1: 500; ab91196; Abcam), and horseradish peroxidase-conjugated anti-rabbit immunoglobulin G (IgG) secondary antibody (1: 1000; ab97051; Abcam). Proteins were visualized using the enhanced chemiluminescence detection kit (Millipore Corp., Millipore Billerica, Massachusetts, United States of America)^[^22^]^.

### Subcellular Localization Analysis

Cytoplasmic and nuclear RNA samples were extracted from cells using the PARIS Kit (Invitrogen). Subsequently, TUG1, U6, and glyceraldehyde-3-phosphate dehydrogenase (or GAPDH) in cytoplasmic and nuclear RNA samples were identified by reverse transcription-quantitative polymerase chain reaction (RT-qPCR).

### Ribonucleic Acid Immunoprecipitation

Possible binding sites between TUG1 and miR-222-3p and argonaute-2 (Ago2) proteins were detected in cells using a ribonucleic acid immunoprecipitation (RIP) kit (17-701; Millipore). Cells at 80% - 90% confluence were centrifuged with radioimmunoprecipitation assay lysis buffer (P0013B). A portion of the cell extract was co-precipitated with the antibody. The bead-antibody mixture was dissolved again in 900 µL of RIP washing buffer and mixed with 100 µL of cell extract. Protein complexes from magnetic beads were gathered, digested with Proteinase K, followed by RNA extraction for RT-qPCR. Antibodies used for RIP were Ago2 (ab32381; 1: 100, Abcam) and anti-human IgG (ab182931; 1: 100, Abcam)^[^23^]^.

### Pull-Down Analysis of Biotinylated Micro Ribonucleic Acids

Sangon (Shanghai, China) was responsible for creating both the biotin-tagged miR-222-3p probe and the control probe. Beads coated with probes were produced through incubation with streptavidin-coated beads (Invitrogen). The cells following lysis were then incubated using the miR-222-3p probe and treated with TRIzol (Takara, Dalian, China) to measure HIF1AN by RT-qPCR^[^24^]^.

### The Luciferase Activity Assay

P19 cells were inoculated into 6-well plates (1.0 × 106 cells/well) and cultured. Cells were transfected with pGL3-TUG1 wild-type (wt)/mutant (mut), pGL3-HIF1AN-3'UTR-wt/mut vectors, and miR-222-3p mimic or mimic NC. Then, cells were assayed using the Dual Luciferase Reporter Gene Assay System (Promega).

### Statistical Analysis

All experimental procedures and result analyses were conducted using a blind method. Data were analyzed using IBM SPSS Statistics for Windows, version 21.0 (IBM Corp., Armonk, N.Y., USA) statistical software. The data were normally distributed as validated by Kolmogorov-Smirnov test, and the results were expressed as mean ± standard deviation. The *t*-test was employed for two-group comparisons, one-way analysis of variance (ANOVA) for multiple groups, and Fisher's LSD-t for pairwise comparisons after ANOVA. Count data were expressed as ratios or percentages, and comparative analyses were performed by chi-square test. *P* denotes a two-sided test; *P* < 0.05 indicates statistical significance.

## RESULTS

### Taurine Up-Regulated Gene 1 and Hypoxia-Inducible Factor 1-Alpha Subunit Inhibitor are Upregulated and miR-222-3p is Downregulated in Ventricular Septal Defect Fetal Mice

**Fig. 1:**
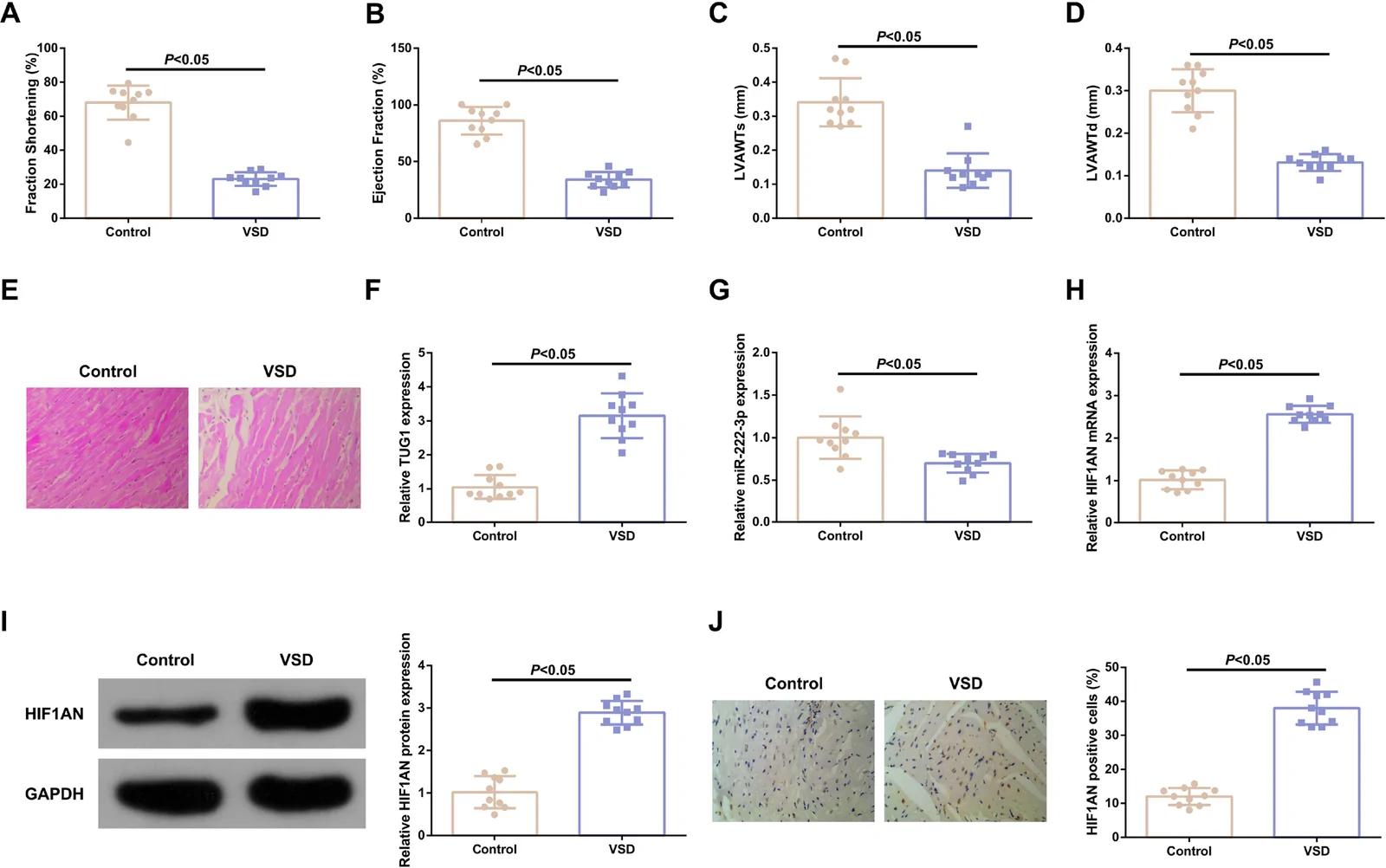
Taurine up-regulated gene 1 (TUG1) and hypoxia-inducible factor 1-alpha subunit inhibitor (HIF1AN) are upregulated and miR-222-3p is downregulated in ventricular septal defect (VSD) fetal mice. A-D) Echocardiography to evaluate cardiac function (left ventricular fractional shortening, ejection fraction, cardiac anterior wall thickness during systole and diastole); E) hematoxylin and eosin staining to detect cardiac tissue pathology; F-I) quantitative polymerase chain reaction and Western Blot to detect TUG1, miR-222-3p, and HIF1AN in cardiac tissue; J) immunohistochemistry staining to detect the positive expression of HIF1AN; n = 10, measurement data were shown as mean ± standard deviation. GAPDH=glyceraldehyde-3-phosphate dehydrogenase; LVAWTd=left ventricular anterior wall thickness at diastole; LVAWTs=left ventricular anterior wall thickness at systole; mRNA=messenger ribonucleic acid.

VPA exposure during pregnancy causes congenital heart disease^[^12^]^. To create an *in vivo* model of VSD, pregnant female mice received an intraperitoneal dose of 700 mg/kg of VPA. Mice cardiac function was evaluated through echocardiography, with attention to LV fractional shortening, ejection fraction, and anterior cardiac wall thickness during systole and diastole (Figure 1A-D). On D19, both VSD and control mice underwent uterine incision delivery procedure, and all fetal hearts were extracted accordingly. As shown in Figure 1E, the interventricular septum and major blood vessels of the control mice developed well, and the atrioventricular valve and aortic valve were mature. VSD, aortic coverage, and dysplasia were observed in VSD mice. Subsequent analysis involved detecting TUG1, miR-222-3p, and HIF1AN in cardiac tissues. In VSD mice, TUG1 and HIF1AN were found to be elevated, whereas miR-222-3p was reduced (Figure 1F-I). HIF1AN positive staining was mainly localized in the cytoplasm, appearing irregular and clay-colored or brown. The positive cells appeared in patchy, diffuse, or focal patterns. In the control group, the heart valve tissue showed pale or sometimes colorless negative staining of HIF1AN, whereas in VSD mice, positive staining of HIF1AN was deep. The positive rate of HIF1AN in the heart valve of VSD mice was elevated (Figure 1J).

### Repression of Taurine Up-Regulated Gene 1 Motivates the Progression and Differentiation of P19 Cells

**Fig. 2:**
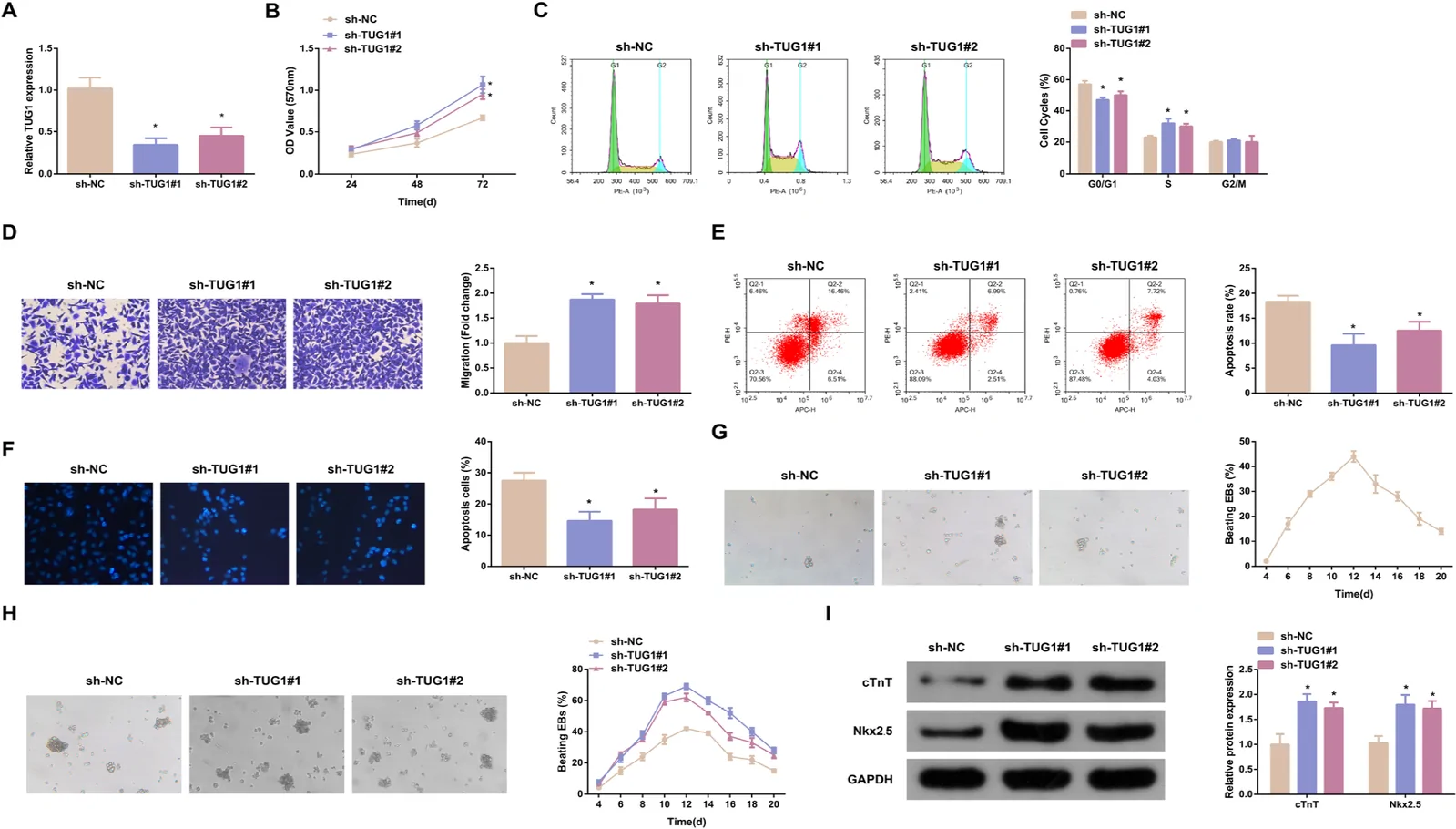
Repression of taurine up-regulated gene 1 (TUG1) motivates the development and differentiation of P19 cells. A) Quantitative polymerase chain reaction detection of TUG1 after repression of TUG1; B) MTT assay to determine cell viability after repression of TUG1; C) flow cytometry detection of cell cycle distribution after repression of TUG1; D) transwell detection of cell migration ability after repression of TUG1; E/F) flow cytometry and Hoechst 33258 to detect the apoptosis of P19 cells after TUG1 repression; G) optical micrographs of dimethylsulfoxide-induced P19 differentiation process at 4, 14, and 20 days; H) P19 cell differentiation ability after TUG1 depression; I) Western Blot to measure cardiac troponin T (cTnT) and Nkx2.5. N = 3. measurement data were shown as mean ± standard deviation. *vs. the short hairpin ribonucleic acid negative control (sh-NC) group, P < 0.05. EBs=embryoid bodies; GAPDH=glyceraldehyde-3-phosphate dehydrogenase; OD=optical density; MTT=3-(4,5-dimethylthiazol-2-yl)-2,5-diphenyltetrazolium bromide; sh-TUG1=short hairpin ribonucleic acid targeting TUG1.

DMSO-induced mouse P19 cells can differentiate into cardiomyocytes *in vitro*, which is a classical model for studying cardiac development, and an important tool for studying cardiomyocyte differentiation^[^25^]^. To explore the underlying molecular mechanisms in VSD, P19 cells were utilized to simulate an *in vitro* model and TUG1 was silenced. To avoid off-target effects, two different TUG1-targeting shRNAs (sh-TUG1#1 and sh-TUG1#2) were applied. Both TUG1 shRNAs can effectively down-regulate TUG1, and sh-TUG1#1 was more effective (Figure 2A). As shown in Fig. 2B-F, TUG1 silencing restrained P19 cell progression. After DMSO induction, P19 cells aggregated into embryoid bodies in a time-dependent manner (Figure 2G). However, in P19 cells treated with TUG1 knockdown, the number of cardiomyocytes induced by DMSO was elevated (Figure 2H). CTnT is a protein specific to cardiomyocytes, indicating late-stage myocardial differentiation. Nkx2.5, one of the first cardiac markers discovered, is mainly located in the heart and cardiac progenitor cells^[^17^]^. Detection of cTnT and Nkx2.5 in cells was conducted, and it was found that both were elevated (Figure 2I).

### Taurine Up-Regulated Gene 1 is a Molecular Sponge of miR-222-3p

**Fig. 3:**
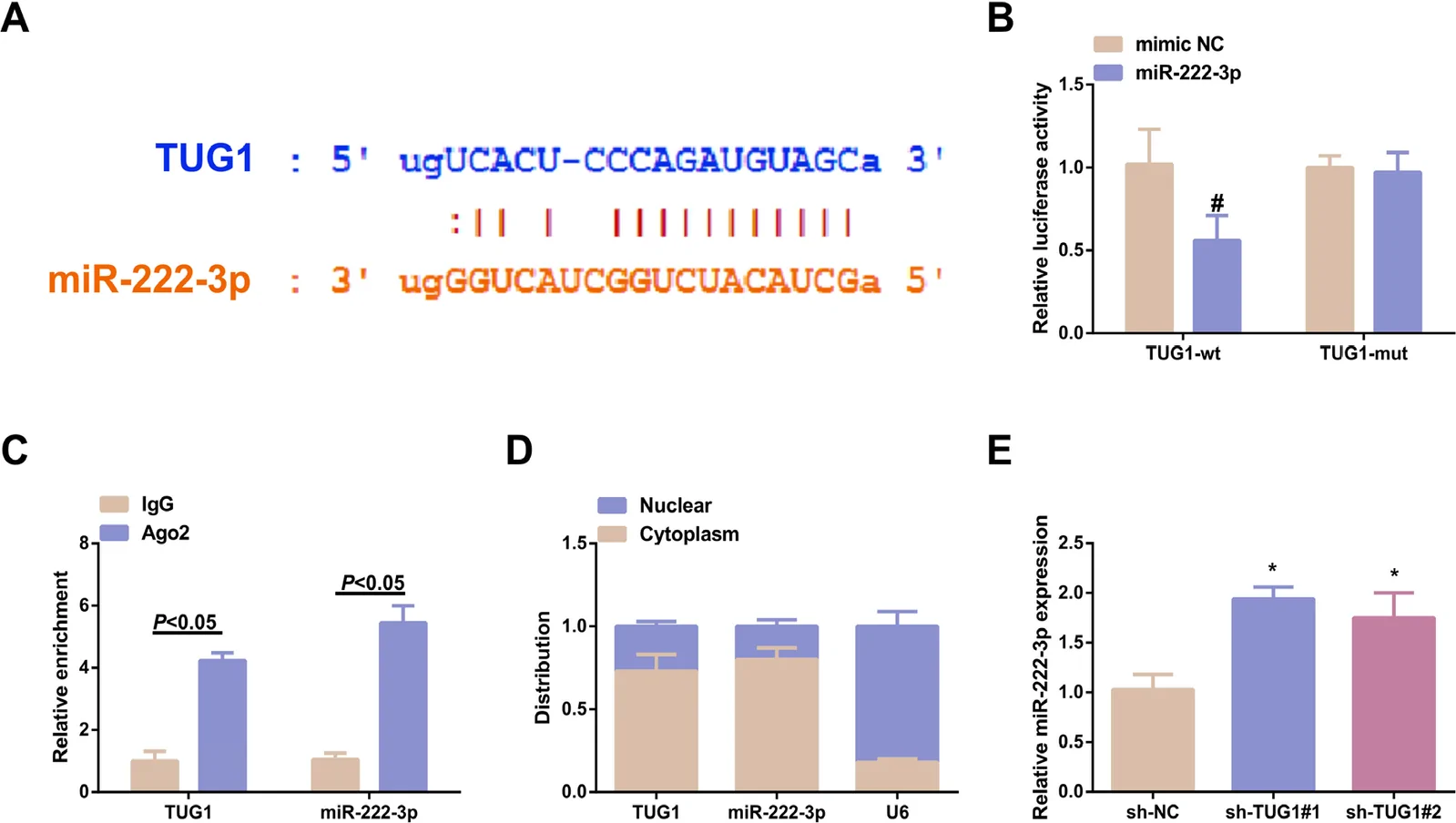
Taurine up-regulated gene 1 (TUG1) is a molecular sponge of miR-222-3p. A) Bioinformatics website (http://starbase.sysu.edu.cn/) to predict TUG1 and miR-222-3p binding sites; B) luciferase activity assay to verify the luciferase activity of miR-222-3p against TUG1; C) ribonucleic acid immunoprecipitation experiments to verify the binding relationship of miR-222-3p to TUG1; D) subcellular localization analysis of TUG1; E) quantitative polymerase chain reaction to detect miR-222-3p after repressing TUG1. N = 3. measurement data were shown as mean ± standard deviation. *vs. the short hairpin ribonucleic acid negative control (sh-NC) group, P < 0.05; #vs. the mimic negative control (NC) group, P < 0.05. Ago2=argonaute-2; IgG=immunoglobulin G; sh-TUG1=short hairpin ribonucleic acid targeting TUG1.

The binding sites of TUG1 and miR-222-3p were predicted on the bioinformatics website (http://starbase.sysu.edu.cn/) (Figure 3A). Furthermore, increased levels of miR-222-3p inhibited the luciferase activity at the TUG1 wild-type binding site, while the mutant binding site remained unaffected (Figure 3B). RIP experiment showed that in P19 cells, TUG1 and miR-222-3p levels in Ago2 precipitates were higher (Figure 3C). Subsequently, subcellular localization analysis clarified that TUG1 was mainly distributed in the cytoplasm of P19 cells (Figure 3D). Meanwhile, it was also found that miR-222-3p level was enhanced after repression of TUG1 in P19 cells (Figure 3E).

### MiR-222-3p Inhibits the Development of P19 Cells and Enhances Their Differentiation into Cardiomyocytes

**Fig. 4:**
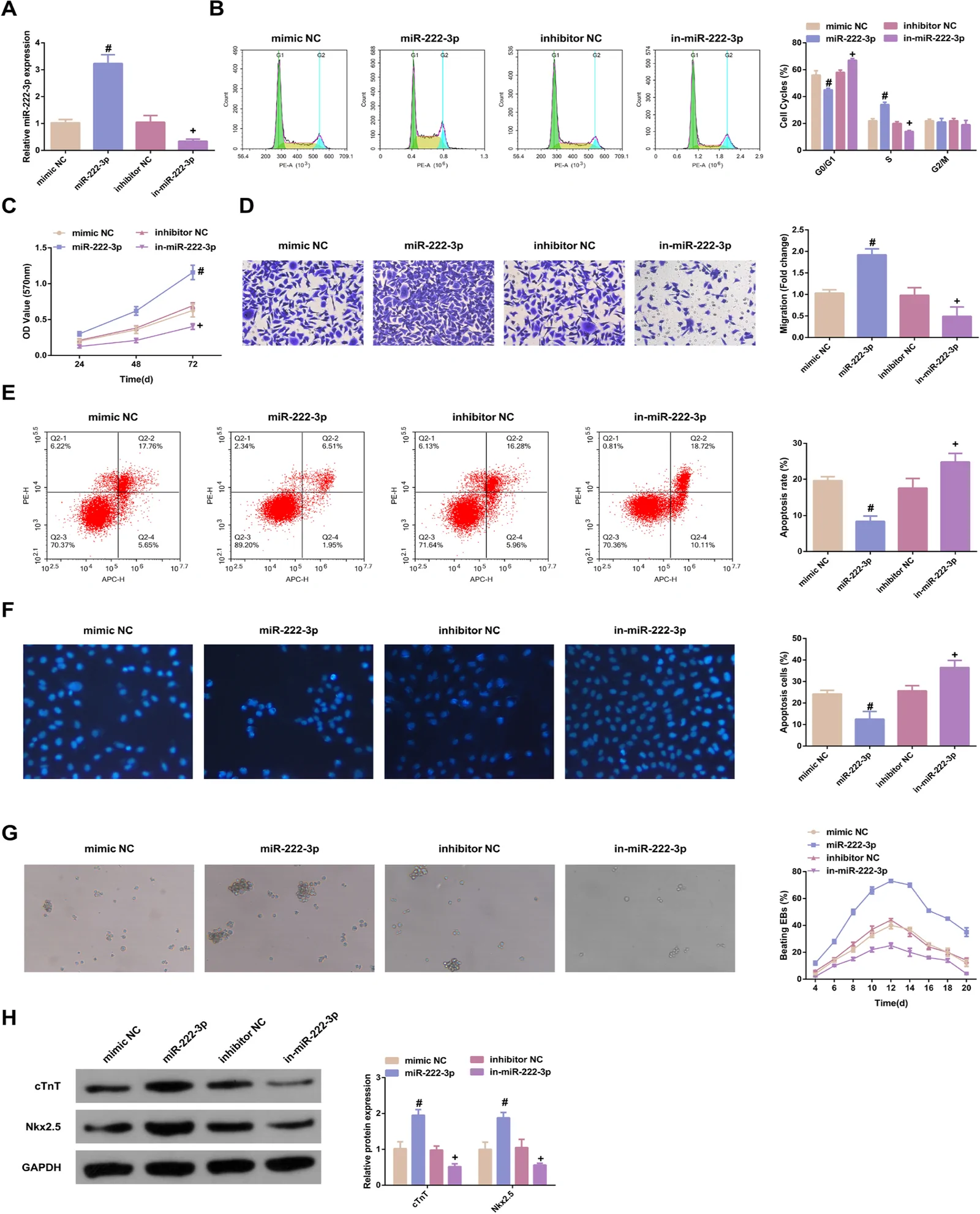
MiR-222-3p depresses P19 cell development and enhances their ability to differentiate into cardiomyocytes. A) Quantitative polymerase chain reaction detection of taurine up-regulated gene 1 after modulation of miR-222-3p; B) MTT assay to determine cell viability after modulation of miR-222-3p; C) flow cytometry detection of cell cycle distribution after modulation of miR-222-3p; D) transwell detection of cell migration ability after modulation of miR-222-3p; E/F) flow cytometry and Hoechst 33258 to detect cell apoptosis after modulation of miR-222-3p; G) P19 cell differentiation ability after modulation of miR-222-3p; H) Western Blot to measure cardiac troponin T (cTnT) and Nkx2.5. N = 3. measurement data were shown as mean ± standard deviation. # P < 0.05, vs. the mimic negative control (NC) group; + P < 0.05, vs. the inhibitor-NC group. EBs=embryoid bodies; GAPDH=glyceraldehyde-3-phosphate dehydrogenase; MTT=3-(4,5-dimethylthiazol-2-yl)-2,5-diphenyltetrazolium bromide; OD=optical density.

P19 cells were transfected with miR-222-3p mimics, inhibitors, and their respective NCs, and the transfection efficiency was tested (Figure 4A). Subsequent experiments manifested that elevation of miR-222-3p motivated P19 cell development (Figure 4B-F). Meanwhile, upregulation of miR-222-3p also elevated the number of cardiomyocytes induced by DMSO (Figure 4G) and elevated cTnT and Nkx2.5 expression (Figure 4H). The results of downregulating miR-222-3p were opposite (Figure 4B-G).

### MiR-222-3p Directly Targets Hypoxia-Inducible Factor 1-Alpha Subunit Inhibitor in P19 Cells

**Fig. 5:**
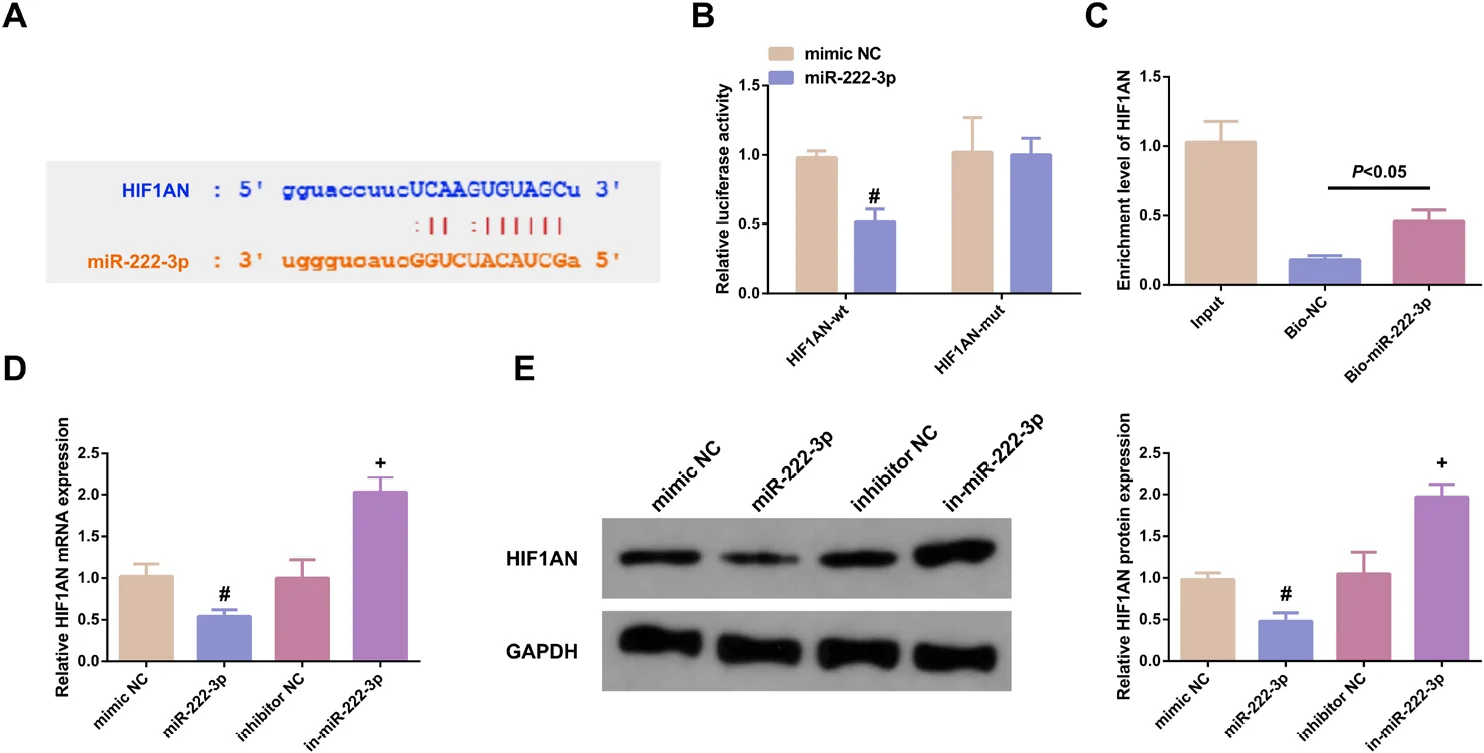
MiR-222-3p directly targets hypoxia-inducible factor 1-alpha subunit inhibitor (HIF1AN) in P19 cells. A) Targeting sequence between miR-222-3p and 3'UTR of HIF1AN in P19 cells; B) luciferase activity detection of the targeting relationship between miR-222-3p and HIF1AN; C) ribonucleic acid pull-down to detect the interaction of miR-222-3p with HIF1AN; D/E) quantitative polymerase chain reaction and Western Blot to analyze HIF1AN after modulation of miR-222-3p. N = 3. measurement data were shown as mean ± standard deviation. # P < 0.05, vs. the mimic negative control (NC) group; + P < 0.05, vs. the inhibitor-NC group. GAPDH=glyceraldehyde-3-phosphate dehydrogenase; mRNA=messenger ribonucleic acid.

Targets of miR-222-3p were predicted using the TargetScan database, with HIF1AN selected as a potential target. Figure 5A shows the targeting sites between miR-222-3p and HIF1AN. The assay for luciferase activity revealed that miR-222-3p's luciferase activity was reduced after co-transfection with the pGL3-3'UTR of HIF1AN, yet it did not change with the pGL3-3'UTR-mut of HIF1AN (Figure 5B). RNA pull-down assay also confirmed that miR-222-3p bound directly to HIF1AN (Figure 5C). Moreover, the results manifested that upregulation of miR-222-3p reduced HIF1AN expression, while downregulation of miR-222-3p enhanced HIF1AN expression (Figure 5D, E).

### Repressing Hypoxia-Inducible Factor 1-Alpha Subunit Inhibitor Motivates P19 Cell Development and Cardiomyocyte Differentiation

**Fig. 6:**
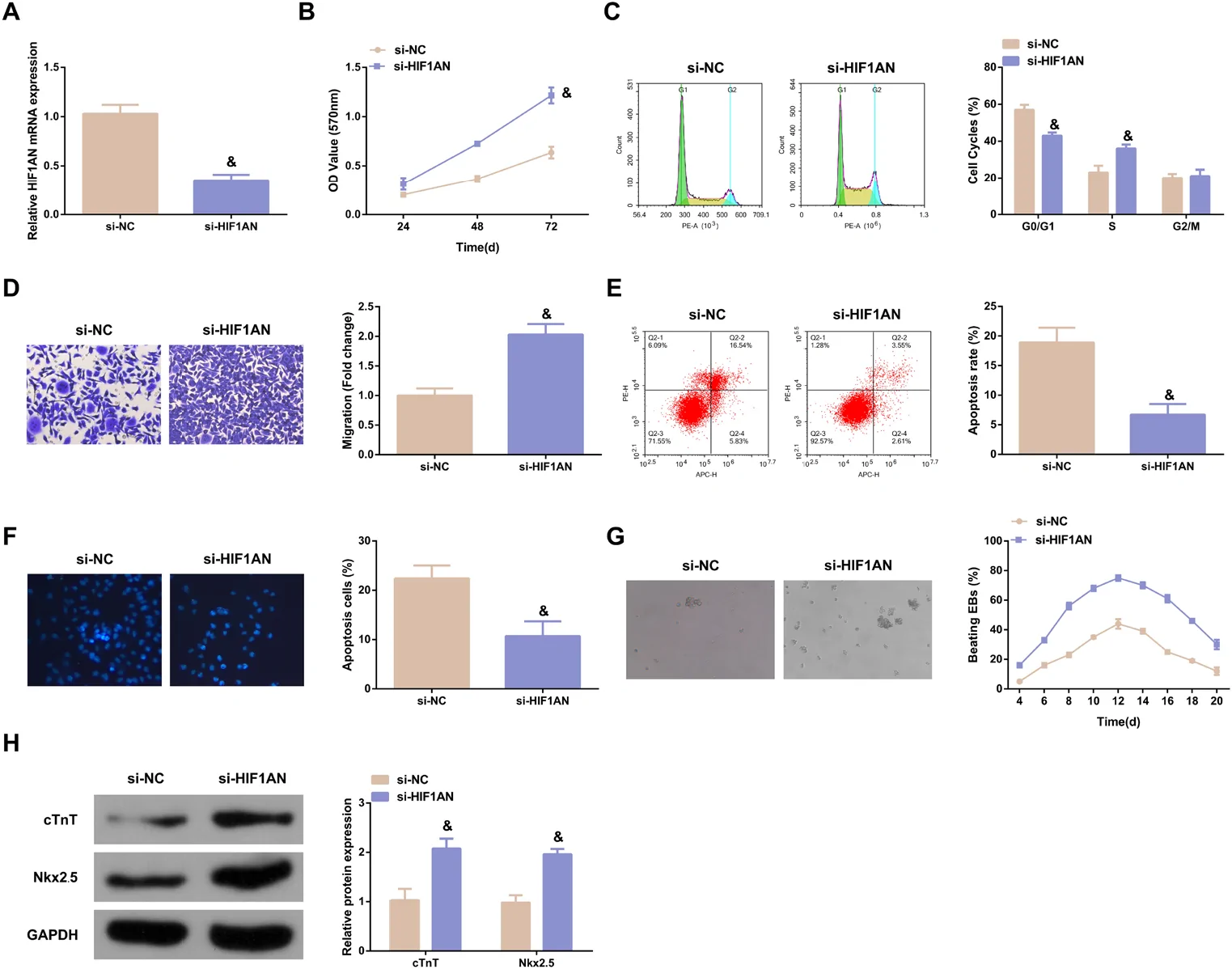
Repressing hypoxia-inducible factor 1-alpha subunit inhibitor (HIF1AN) motivates P19 cell development and cardiomyocyte differentiation. A) Quantitative polymerase chain reaction detection of HIF1AN after repression of HIF1AN; B) MTT assay to determine cell viability after repression of HIF1AN; C) flow cytometry detection of cell cycle distribution after repression of HIF1AN; D) transwell detection of cell migration ability after repression of HIF1AN; E/F) flow cytometry and Hoechst 33258 to detect cell apoptosis after HIF1AN repression; G) P19 cell differentiation ability after HIF1AN depression; I) Western Blot to measure cardiac troponin T (cTnT) and Nkx2.5. N = 3. measurement data were shown as mean ± standard deviation. & vs. the si-NC group, P < 0.05. EBS=embryoid bodies; GAPDH=glyceraldehyde-3-phosphate dehydrogenase; mRNA=messenger ribonucleic acid; MTT=3-(4,5-dimethylthiazol-2-yl)-2,5-diphenyltetrazolium bromide; NC=negative control; OD=optical density.

After knockdown of HIF1AN in P19 cells, it was found that HIF1AN expression was reduced (Figure 6A). Knockdown of HIF1AN resulted in enhanced P19 cell development (Figure 6B-F). Meanwhile, depressing HIF1AN also increased the number of cardiomyocytes induced by DMSO (Figure 6G) and increased cTnT and Nkx2.5 expression (Figure 6H).

### Hypoxia-Inducible Factor 1-Alpha Subunit Inhibitor Elevation Reverses the Functions of Downregulating Taurine Up-Regulated Gene 1 or Upregulating miR-222-3p on P19 Cells

**Fig. 7:**
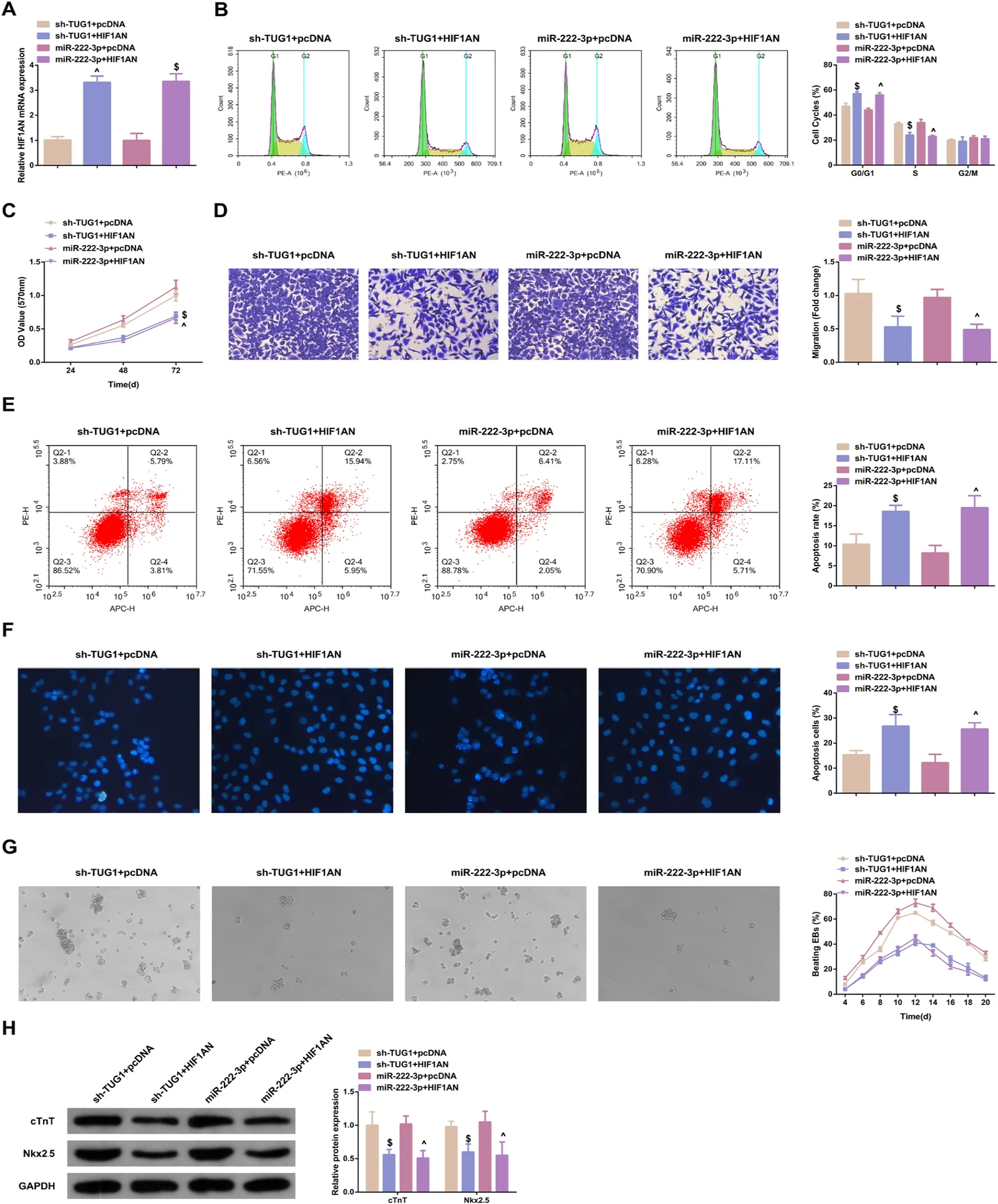
Hypoxia-inducible factor 1-alpha subunit inhibitor (HIF1AN) elevation reverses the functions of repression of taurine up-regulated gene 1 (TUG1) or elevation of miR-222-3p on P19 cells. A) Quantitative polymerase chain reaction detection of HIF1AN; B) MTT assay to determine cell viability; C) flow cytometry detection of cell cycle distribution; D) transwell detection of cell migration ability; E/F) flow cytometry and Hoechst 33258 to detect cell apoptosis; G) P19 cell differentiation ability; H) Western Blot to measure cardiac troponin T (cTnT) and Nkx2.5. N = 3. measurement data were shown as mean ± standard deviation. ^ vs. the sh-TUG1=short hairpin ribonucleic acid targeting TUG1 (sh-TUG1)+plasmid cloning deoxyribonucleic acid (pcDNA) group, P < 0.05; $ vs. the miR-222-3p+pcDNA group, P < 0.05. APC-H=allophycocyanin-height; EBS=embryoid bodies; GAPDH=glyceraldehyde-3-phosphate dehydrogenase; mRNA=messenger ribonucleic acid; MTT=3-(4,5-dimethylthiazol-2-yl)-2,5-diphenyltetrazolium bromide; OD=optical density; PE-H=phycoerythrin-height.

To further investigate the molecular mechanism of TUG1/miR-222-3p/HIF1AN in P19 cells, HIF1AN was overexpressed in P19 cells transfected with shTUG1 or miR-222-3p mimic (Figure 7A). Elevating HIF1AN counteracted the effects of TUG1 inhibition or miR-222-3p overexpression on P19 cells, suppressing cell development, reducing the number of P19 cells differentiating toward cardiomyocytes, and decreasing both cTnT and Nkx2.5 expression (Figure 7B-H).

## DISCUSSION

Although VSD is a widely recognized type of CHD, its underlying mechanisms remain uncertain. LncRNAs are being recognized as important in VSD, with more evidence supporting this, and examining their regulatory mechanisms can help elucidate the pathogenesis of VSD. In this study, it was found that TUG1 and HIF1AN were elevated but miR-222-3p was reduced in cardiac tissues of VSD mice. *In vitro*, repression of TUG1 and HIF1AN or elevation of miR-222-3p facilitated P19 cell development and cardiomyocyte differentiation. TUG1 accelerated VSD formation in children by targeting HIF1AN via miR-222-3p.

LncRNAs have important functions in cardiac development. Song et al.^[^26^]^ reported a total of 880 lncRNAs that are up-regulated and 628 lncRNAs that are reduced in VSD. Cheng et al.^[^27^]^ reported that lncRNA TUC40 is elevated in VSD, and elevation of TUC40 stimulates cellular differentiation and apoptosis while repressing cellular proliferation^[^28^]^. Wu et al.^[^29^]^ reported that lncRNA-uc.40 is highly expressed in abnormal human embryonic hearts, and silencing lncRNA-uc.40 facilitates the differentiation of P19 embryonic cells into cardiomyocytes by targeting PBX1. This study mainly explored the biological function of TUG1 in VSD. Exposure to VPA during pregnancy has been reported to result in CHD^[^12^]^. Thus, a VSD model was established by injecting VPA into pregnant mice intraperitoneally. The findings indicated that TUG1 levels were higher in VSD mice, implying a role for TUG1 in heart development. *In vitro* results showed that knockdown of TUG1 promoted the development of P19 cells. Exploring the molecular basis of embryonic stem cell differentiation into cardiomyocytes may offer insights into effective cardiovascular disease therapies^[^30^]^. This study revealed that silencing TUG1 encouraged P19 cells to differentiate into cardiomyocytes, as evidenced by a significant elevation in the cardiomyocyte-specific markers cTnT and Nkx2.5. TUG1 contributes to particulate matter (or PM) exposure-induced airway hyperresponsiveness by absorbing miR-222-3p^[^31^]^. Likewise, TUG1 was shown to play a role in VSD by absorbing miR-222-3p.

Differential expression of miR-222-3p has been observed in mouse and human cardiac pathology^[^32^]^. MiR-222-3p expression is reduced in hypoxic cardiomyocytes, and elevation of miR-222-3p facilitates cell proliferation but refrains apoptosis. miR-222-3p has been testified to be reduced in VSD, however its biological function has not been elucidated^[^11^]^. Here, it was also found that miR-222-3p was reduced in VSD. Meanwhile, elevation of miR-222-3p promoted P19 cell development and differentiation and elevated intracellular cTnT and Nkx2.5, whereas inhibition of miR-222-3p did the opposite.

It is well known that hypoxia is a common feature of patients with coronary artery disease^[^33^]^. Hypoxia-inducible factor 1-alpha (HIF-1α) is a well-recognized master regulator of cellular defense mechanisms against hypoxic stress and has been shown to be associated with coronary heart disease development^[^34^]^. HIF1AN, a negative modulator of HIF-1α, is up-regulated in the cardiomyocyte hypoxia-reoxygenation (H/R) model, and its expression is modulated by miRNAs. Knockdown HIF1AN suppresses the effects of H/R on cardiomyocytes and represses the release of apoptosis and inflammatory cells factors. These data suggest that HIF1AN is likely involved in the development of CHD. In VSD, HIF1AN was found to be elevated and showed a negative correlation with miR-222-3p expression. It was verified through TargetScan and luciferase activity assays that HIF1AN is a downstream target of miR-222-3p. Meanwhile, HIF1AN knockdown enhanced P19 cell development, differentiation, and intracellular cTnT and Nkx2.5 expression, while HIF1AN overexpression reversed the functions of silencing TUG1 or enhancing miR-222-3p, implying that the TUG1/miR-222-3p /HIF1AN axis plays an important role in the formation of VSD.

### Limitations

This study still has some limitations. First, cardiac development is a complex process that involves multiple cells and signaling pathways. The P19 cell model has limitations in reflecting the full picture of embryonic cardiac development. The function of TUG1 in cardiac development needs to be further verified. Secondly, HIF1AN is a negative regulator of HIF-1α, and the effect of knockdown/elevation of TUG1/miR-222-3p on HIF-1α and the interaction between the two still need to be further investigated.

## CONCLUSION

This study ultimately confirmed that TUG1 contributes to VSD by targeting HIF1AN via miR-222-3p. The study sheds light on the involvement and regulation of lncRNAs in VSD, proposing TUG1 as a potential novel target for VSD therapy.

## Data Availability

The authors declare that the data will be available upon reasonable request to the authors.

## Artificial Intelligence Usage

The authors declare that no artificial intelligence tool was used in the preparation of this article.
